# Gut-lung axis perturbation and *Bifidobacterium* potential after spinal cord injury in humans and mice

**DOI:** 10.1016/j.isci.2026.114655

**Published:** 2026-01-09

**Authors:** Yuanqing Ding, Xingyu Chen, Yiming Tao, Haoru Dong, Zezhen Zhang, Xiao Xiao, Gong Chen, Xiaomu Li, Rong Xie

**Affiliations:** 1 Department of Neurosurgery, National Center for Neurological Disorders, Neurosurgical Institute of Fudan University, Shanghai Clinical Medical Center of Neurosurgery, Shanghai Key Laboratory of Brain Function and Restoration and Neural Regeneration, Huashan Hospital, Fudan University, Shanghai 200040, P.R. China; 2 Department of Endocrinology, The First Affiliated Hospital, Zhejiang University School of Medicine, Hangzhou 310003, P.R. China; 3 Department of Neurosurgery, Neurosurgery Research Institute, Clinical Research and Translation Center, National Regional Medical Center, the First Affiliated Hospital, Fujian Medical University, Fuzhou, Fujian Province 350035, P.R. China; 4 Department of Neurosurgery, Tongren Municipal People’s Hospital, Tongren, Guizhou Province 554300, P.R. China

**Keywords:** health sciences

## Abstract

Spinal cord injury (SCI) predisposes patients to severe respiratory complications. Integrating clinical observations with a mouse model, we identified gut-lung axis perturbation as a key mechanism. SCI increased the similarity between gut and lung microbial communities, consistent with cross-compartment convergence driven by intestinal dysbiosis. Fecal microbiota transplantation from SCI donors reproduced this convergence in healthy recipients. Crucially, oral *Bifidobacterium* supplementation restored intestinal community structure, reduced gut-lung similarity, and attenuated pulmonary inflammation following an *Escherichia coli* challenge. These findings indicate that SCI disrupts gut-lung compartmentalization and that augmenting beneficial gut taxa mitigates downstream pulmonary consequences. Microbiota-targeted strategies therefore warrant evaluation as adjuncts to reduce post-SCI respiratory risk.

## Highlights

• SCI increases gut-lung microbiota similarity in humans and mice
• Source tracking supports post-SCI cross-compartment convergence
• Oral *Bifidobacterium* improves gut ecology and lowers gut-lung similarity
• Probiotic treatment attenuates lung inflammation after *E. coli* challenge in mice

## Graphical abstract

**Figure:**
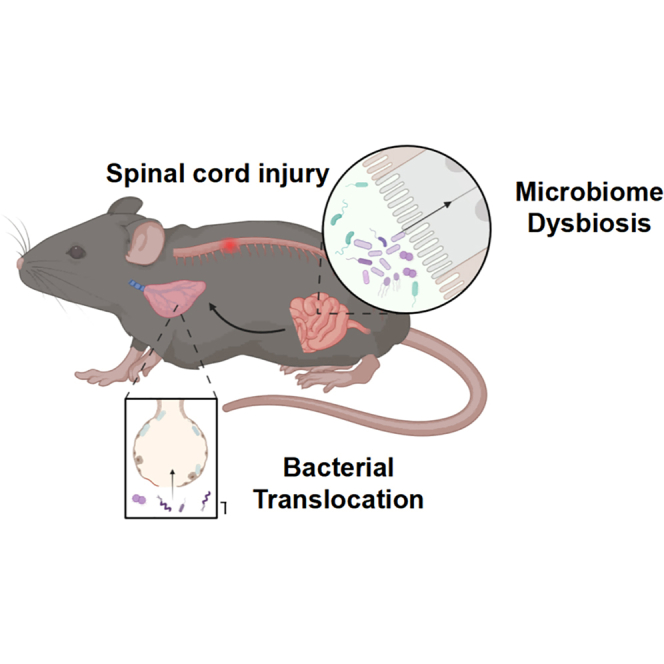

## Introduction

Spinal cord injury (SCI) is a life-altering condition that causes persistent neuromotor deficits and a heavy burden of chronic complications. Among these, infection—most notably pneumonia—remains a leading cause of morbidity and mortality, far exceeding general-population baselines.^1^^,^^2^^,^^3^ Post-SCI pneumonia is clinically complex and independently predicts worse neurological recovery.^4^^,^^5^ Cervical and high-thoracic injuries amplify risk by impairing ventilatory mechanics and cough, predisposing to atelectasis, secretion retention, aspiration, and ventilator dependence.^6^^,^^7^^,^^8^ In parallel, SCI-induced immunodepression (SCI-IDS) is characterized by blunted innate and adaptive responses driven, in part, by sympathetic overactivation and hypothalamic-pituitary-adrenal axis perturbation, weakening pulmonary host defenses.^9^^,^^10^^,^^11^

Beyond direct respiratory impairment and systemic immune dysfunction, emerging work highlights the gut-lung axis as a third pathway shaping post-SCI pulmonary vulnerability.^12^^,^^13^ The gut, the body’s largest microbial reservoir, orchestrates systemic immune tone through microbe-derived metabolites and mucosal signaling.^14^ The lungs, though low-biomass, harbor resident microbial communities that contribute to local immunity and colonization resistance.^12^^,^^15^^,^^16^ SCI disrupts bowel motility and autonomic regulation, leading to gut dysbiosis and barrier dysfunction.^17^^,^^18^^,^^19^^,^^20^ In murine T9 models, SCI has been associated with dysbiosis accompanied by detection of gut-associated taxa or signals within the blood.^21^^,^^22^^,^^23^^,^^24^^,^^25^^,^^26^^,^^27^ Mechanistically, dysbiosis and barrier compromise may facilitate translocation of bacteria or their products and amplify inflammatory responses to subsequent respiratory challenges.^22^^,^^23^^,^^24^^,^^25^^,^^26^^,^^27^

Despite these advances, the magnitude and clinical relevance of gut-lung crosstalk after SCI remain insufficiently defined in humans, and putative mechanisms such as bacterial translocation are not fully validated. We therefore sought to map microbiome changes across gut and lung compartments after SCI and to test whether targeted modulation of gut communities can mitigate downstream pulmonary consequences.

We hypothesized that SCI perturbs the gut-lung axis by increasing the similarity between gut and lung microbial communities—consistent with enhanced gut-derived signals in the airways—and that restoring intestinal balance with a probiotic would attenuate this convergence and reduce pulmonary inflammation. To address this, we integrated observations from patients with SCI and a T9-transection mouse model. We profiled gut and lung microbiota using 16S rRNA gene amplicon sequencing in both species and used source-tracking analyses to estimate cross-compartment contributions. To probe causality, we performed fecal microbiota transplantation (FMT) from SCI donor mice to healthy recipients. Finally, we administered oral *Bifidobacterium longum* to SCI mice and challenged them with GFP-expressing *Escherichia coli (E. coli)* to test effects on gut barrier integrity, gut-to-lung microbial signals, and airway inflammation. We demonstrate that SCI disrupts the gut-lung axis and that augmenting beneficial taxa such as *Bifidobacterium* can mitigate these changes in experimental models, motivating evaluation of microbiota-targeted adjuncts to lower post-SCI respiratory risk.

## Results

### SCI is associated with increased pneumonia-related symptoms and altered sputum microbiota

**Figure 1:**
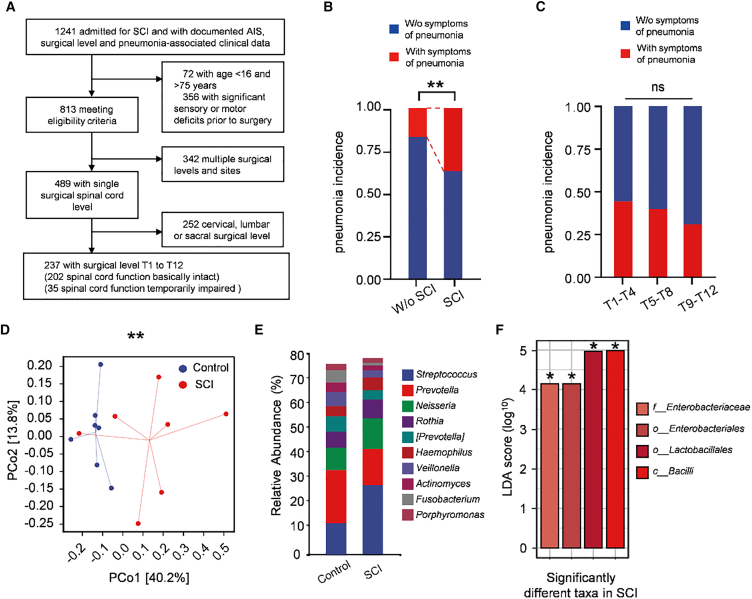
Alterations in the pulmonary microbiota in patients with SCI (A) Flowchart of the retrospective cohort. Adults undergoing thoracic spinal surgery between 2019 and 2022 at Huashan Hospital were screened; 237 patients with surgical levels T1–T12 met eligibility criteria. (B) Proportion of patients with pneumonia-related symptoms with and without SCI. Patients with SCI showed a higher frequency (χ^2^ test, *p* = 0.009; without SCI, *n* = 202; SCI, *n* = 35). (C) Incidence of pneumonia-related symptoms by thoracic surgical level (T1–T4, T5–T8, T9–T12); no significant differences were observed (χ^2^ test, *p* = 0.822; *n* = 10, 9, 16, respectively; ns). (D) PCoA of weighted UniFrac distances showing separation of sputum communities between groups (PERMANOVA, *p* = 0.008; control, *n* = 7; SCI, *n* = 7). (E) Genus-level taxonomic composition of sputum by 16S rRNA gene sequencing (control, *n* = 7; SCI, *n* = 7). (F) LEfSe linear discriminant analysis（LDA) score bar plot (threshold LDA>4) highlighting taxa differentially abundant between SCI and control.

We analyzed 237 consecutive patients who underwent thoracic spinal surgery between 2019 and 2022 according to prespecified eligibility criteria (Figure 1A). Baseline characteristics (age and sex) were comparable between patients with preserved thoracic cord function and those with postoperative thoracic cord dysfunction (age: *p* = 0.396; sex: *p* = 0.170; Table S1). Thirty-five patients met criteria for postoperative thoracic cord dysfunction (American Spinal Injury Association [ASIA] Impairment Scale grade A–D), a clinical picture resembling acute traumatic SCI. Pneumonia-related symptoms occurred more frequently in patients with thoracic cord dysfunction than in those with preserved function (37.1% vs. 17.3%, *p* = 0.009; Figure 1B). Stratification by injury level (high T1–T4, mid T5–T9, low T10–T12) did not reveal differences in symptom incidence (*p* = 0.822; Figure 1C and Table S2), suggesting that dysfunction at any thoracic level may increase pulmonary vulnerability.

To profile postoperative lung community structure, we prospectively enrolled 14 thoracic-surgery patients and collected sputum 1 week after surgery for 16S rRNA gene sequencing. Groups were defined by postsurgical neurological status (control, *n* = 7; SCI, *n* = 7). Weighted UniFrac principal coordinate analysis (PCoA) showed clear separation between groups (PERMANOVA, *p* = 0.008; Figure 1D). At the genus level, the group with SCI exhibited increased *Streptococcus* and decreased *Prevotella* (Figure 1E), taxa respectively linked to higher post-SCI respiratory risk and a more favorable pulmonary inflammatory tone.^28^^,^^29^^,^^30^ Linear discriminant analysis effect size (LEfSe) identified enrichment of gut-associated orders (*Enterobacterales* [LDA score = 4.15, *p* = 0.034], *Lactobacillales* [LDA score = 4.91, *p* = 0.048]) and higher abundance of *Enterobacteriaceae* (LDA score = 4.15, *p* = 0.034) and the class Bacilli (LDA score = 4.99, *p* = 0.048) in the SCI group (Figure 1F). Collectively, these data indicate that thoracic SCI is associated with increased pneumonia-related symptoms and a reshaped lung microbiota featuring enrichment of gut-associated taxa, consistent with enhanced gut-derived signals in the airways.

### SCI induces subacute pulmonary inflammation in mice

**Figure 2:**
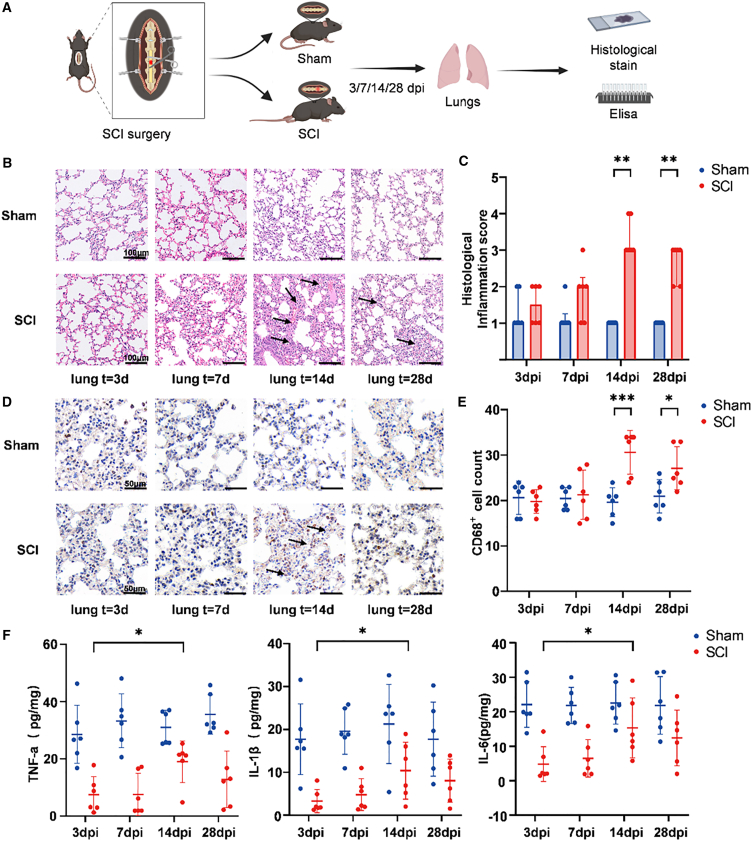
SCI induces subacute lung inflammation in mice (A) Experimental timeline. Lungs were collected at 3, 7, 14, and 28 dpi for histology and ELISA quantification. (B) Representative H&E-stained lung sections from Sham and SCI mice at 3, 7, 14, and 28 dpi (scale bars, 100 μm; *n* = 6 per group per time point). (C) Histological inflammation scores. Comparisons between Sham and SCI at each time point (two-tailed Mann-Whitney test; Median ± IQR; ∗∗*p* < 0.01; *n* = 6 per group). (D) Representative immunohistochemistry for CD68^+^ cells in lungs at 3, 7, 14, and 28 dpi (scale bars, 50 μm; black arrows indicate perivascular/peribronchial infiltrates; *n* = 6 per group per time point). (E) Quantification of CD68^+^ cells (mean ± SD). Comparisons between Sham and SCI at each time point (unpaired two-tailed *t* test; ∗*p* < 0.05, ∗∗∗*p* < 0.001; *n* = 6 per group). (F) ELISA measurements of TNF-α, IL-1β, and IL-6 in lung tissue (mean ± SD). Comparison within the SCI group between 3 and 14 dpi (unpaired two-tailed *t* test; ∗*p* < 0.05; *n* = 6 per group).

To determine whether SCI perturbs the pulmonary milieu, we performed T9 complete transection in specific pathogen-free (SPF) mice and sampled lungs at 3, 7, 14, and 28 days post-injury (dpi) (Figure 2A). Hematoxylin and eosin (H&E) staining in sham-operated mice showed preserved alveolar architecture without inflammation, whereas SCI produced perivascular and peribronchial leukocytic infiltrates evident by 14 dpi (*p* = 0.0022) and persisting at 28 dpi (*p* = 0.0022) (Figures 2B and 2C). Morphometric analyses of the alveolar space/pulmonary parenchyma ratio, an indicator of septal thickness and interstitial edema, showed a significant reduction in the SCI group compared to Sham. This difference was evident at 7 (*p* = 0.0334), 14 (*p* = 0.0005), and 28 dpi (*p* = 0.0067; Figures S1A and S1B). We next assessed interstitial edema, which showed a significant increase in the SCI group compared to Sham exclusively at 14 dpi (*p* = 0.0014; Figures S1C and S1D). Immunohistochemistry revealed a significant increase in the number of CD68^+^ myeloid cells in SCI lungs at 14 dpi (*p* = 0.0011) and 28 dpi (*p* = 0.0118) (Figures 2D and 2E), and bronchoalveolar lavage total cell counts showed a significant, transient increase in the SCI group exclusively at 14 dpi (*p* = 0.0306) (Figure S1E), consistent with a transient phase of enhanced leukocyte recruitment.^31^

To contextualize these histological changes, we quantified lung cytokines at 14 dpi. In line with SCI-IDS, proinflammatory cytokines were overall lower in SCI than in Sham.^9^ Nonetheless, within the SCI group, TNF-α, IL-1β, and IL-6 increased from 3 to 14 dpi (*p* = 0.0481, *p* = 0.0461, *p* = 0.0443) (Figure 2F), indicating a subacute rebound in pulmonary inflammatory signaling. Systemic changes accompanied the lung phenotype. Specifically, splenic lymphoid follicles were enlarged at 14 dpi (Figure S2A), and complete blood counts were altered. Within the SCI group, total white blood cell (WBC) counts were significantly increased from the 3 dpi baseline at 14 dpi (*p* = 0.0045) and remained elevated at 28 dpi (*p* = 0.0361) (Figure S2B). This was accompanied by a significantly higher percentage of neutrophils in the SCI group at 14 dpi (*p* = 0.0019) (Figure S2D).

### Disruption of the lung microbiota in mice

**Figure 3:**
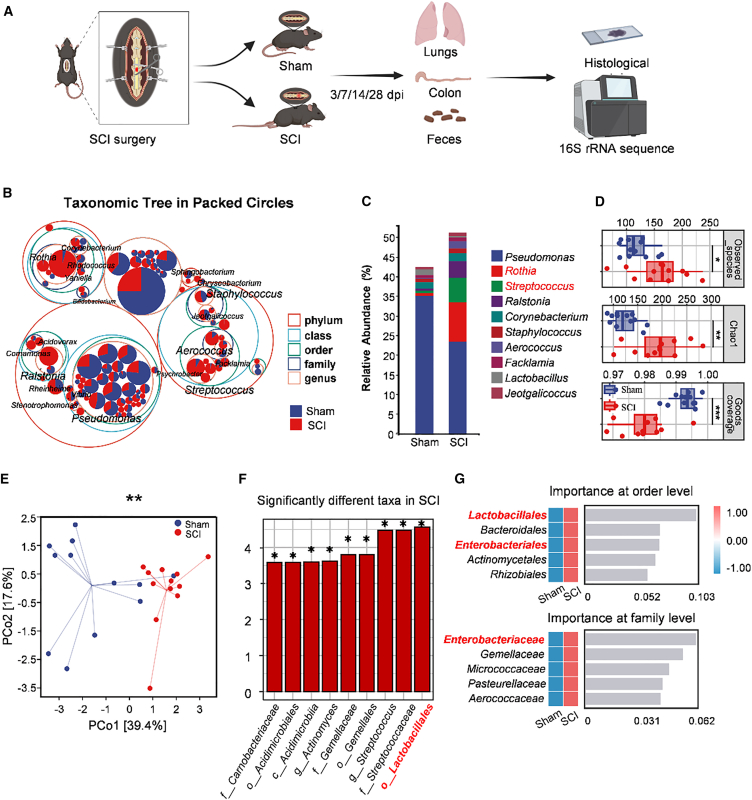
Differential lung microbiota composition in SCI versus Sham mice (A) Experimental flowchart. Fecal, lung, and intestinal samples were collected from the SCI or Sham group at 14 dpi for 16S rRNA sequencing and histological examination. (B) Circle-packing taxonomic tree of lung microbiota comparing Sham and SCI. (C) Genus-level taxonomic composition of lung microbiota (*n* = 12 per group). (D) Analysis of alpha diversity metrics revealed variations in microbial diversity between the SCI and Sham groups. Compared with the Sham group, the Chao1 and observed_species metrics in the SCI group were significantly greater, and Good’s coverage was significantly lower. ∗*p* < 0.05; ∗∗*p* < 0.01; ∗∗∗*p* < 0.001 for the SCI group versus the sham group (Kruskal‒Wallis test; *n* = 12; error bars represent the means ± interquartile ranges [IQRs]). (E) PCoA based on weighted UniFrac distances clearly revealed separation between the microbiotas of Sham and SCI mice (PERMANOVA; ∗∗*p* < 0.01; *n* = 12 per group). (F) LEfSe LDA score bar plot (threshold LDA > 3) highlighting taxa differentially abundant in SCI versus Sham. (G) Random forest feature importance at the order and family levels for discriminating SCI from Sham.

We profiled lung communities by 16S rRNA gene amplicon sequencing in SPF mice subjected to T9 transection (SCI) or sham surgery (Sham) (Figure 3A). A circle-packing taxonomic tree provided a hierarchical overview and highlighted enlarged nodes for *Streptococcus* and *Staphylococcus*, with increases in mucosa-associated commensals such as *Rothia* and *Aerococcus* in SCI lungs (Figure 3B). Consistently, genus-level bar plots showed higher relative abundances of these taxa in SCI than in Sham (Figure 3C).

Alpha diversity indices increased in SCI compared with Sham, as shown by significant differences in Chao1 (*p* = 0.0015), observed_species (*p* = 0.021), and goods_coverage (*p* = 0.00031). This indicates greater within-sample diversity and the appearance of additional taxa in the injured state (Figure 3D). Beta diversity based on weighted UniFrac distances showed a significant compositional shift between groups, confirmed by a permutational multivariate analysis of variance (PERMANOVA; pseudo-F = 7.79, *p* = 0.001). This separation was visualized on a PCoA ordination (Figure 3E). LEfSe identified enrichment of gut- and oral-associated lineages in SCI lungs, including the order Lactobacillales (LDA score = 4.56, *p* = 0.0292), a finding consistent with the LEfSe data from clinical patients (Figures 3F and 1E). A random forest classifier ranked *Lactobacillales* and *Enterobacterales* (order level) and *Enterobacteriaceae* (family level) as top features distinguishing SCI from Sham (Figure 3G). These murine alterations mirror human sputum patterns (Figure 1E), supporting disruption of the gut-lung axis after SCI.

### SCI induces gut microbiota alterations and increased gut-lung convergence

**Figure 4:**
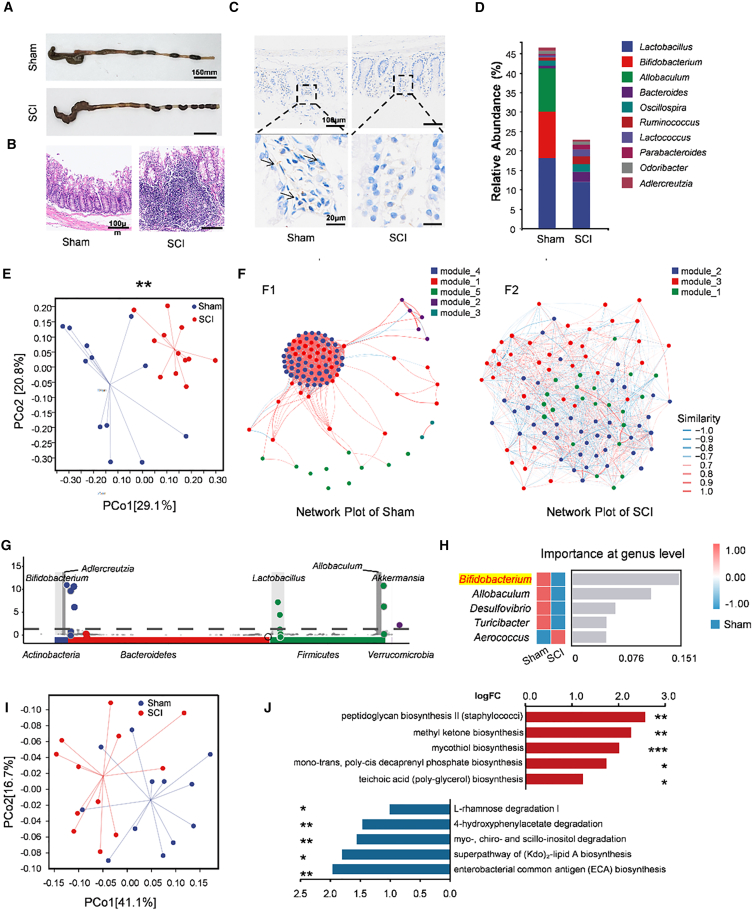
Changes in intestinal structure and gut microbiome dysfunction in SCI (A) Representative gross anatomy of the colon. Compared with the Sham group, fecal accumulation was greater in the SCI group. scale bar, 150 mm. (B) Representative H&E-stained cross sections of the colon (scale bars, 100 μm; *n* = 12). (C) Representative images of immunohistochemical staining for PGP9.5 in the colons of SCI and Sham mice. Arrows indicate positively stained cells (scale bars, 100 and 20 μm; *n* = 12). (D) Taxonomic composition of the gut microbiota at the genus level in the SCI and Sham groups (*n* = 12). (E) PCoA based on weighted UniFrac distances demonstrating distinct clustering patterns between the gut microbiota of the SCI and Sham groups (PERMANOVA; ∗∗*p* < 0.01; *n* = 12 per group). (F) Network plot comparing the gut microbiota networks of Sham (F1) and SCI (F2) mice (*n* = 12). (G) MetagenomeSeg analysis revealed the core microbiota present in the Sham group compared with the SCI group. (H) Random forest analysis of differences in the taxonomic composition of the gut microbiota between SCI and Sham groups at the genus level. (I) PCoA based on MetaCyc metabolic pathway abundances showing distinct functional clustering between Sham and SCI groups. (J) Functional prediction of gut microbiota metabolic pathways (MetaCyc) using PICRUSt2. Bar plot shows the top 10 differentially abundant pathways. Red bars indicate pathways enriched in the SCI group, while blue bars indicate pathways enriched in the Sham group (two-sided Welch’s *t* test; ∗*p* < 0.05, ∗∗*p* < 0.01, ∗∗∗*p* < 0.001).

Given the enrichment of gut- and oral-associated taxa in the lung after SCI (Figures 1E, 3B, and 3C), we next examined intestinal function and community structure at 14 dpi. Gross anatomy revealed marked fecal retention and a shortened colon in SCI mice, consistent with impaired motility (Figure 4A).^32^ H&E staining showed mucosal and submucosal inflammation (Figure 4B). PGP9.5 immunohistochemistry, a pan-neuronal marker of the enteric nervous system,^33^^,^^34^ demonstrated reduced immunoreactivity in SCI versus Sham, indicating loss of enteric innervation (Figure 4C).

Genus-level composition of fecal/colonic samples showed depletion of *Lactobacillus*, *Bifidobacterium*, and *Allobaculum* in SCI relative to Sham (Figure 4D). Weighted UniFrac PCoA demonstrated a compositional shift between groups (Figure 4E). Co-occurrence network analysis revealed a loss of ecosystem stability in SCI. The Sham network was robust, characterized by 5 modules and a high transitivity (clustering coefficient) of 0.8885. In contrast, the SCI network was fragmented, with only 3 modules and a significantly lower transitivity of 0.6029 (Figure 4F).

Differential abundance testing (metagenomeSeq) identified a Sham-enriched core with significant depletion of *Bifidobacterium* in SCI (log_2_ fold change = 6.705, *p* < 0.0001; Figure 4G). A genus-level random forest classifier likewise ranked reduced *Bifidobacterium* among the top features distinguishing SCI from Sham (Figure 4H). To determine whether these compositional changes corresponded to functional shifts, we performed predictive functional profiling using PICRUSt2. PCoA of metabolic pathways (MetaCyc) revealed distinct functional clustering between the SCI and Sham groups (Figure 4I). Crucially, the SCI group exhibited a significant enrichment of pathways associated with pathogen virulence and cell wall synthesis, including peptidoglycan biosynthesis II and teichoic acid biosynthesis (Figure 4J). These functional alterations suggest a shift toward a pro-inflammatory and pathogenic microbial community. Together, these data indicate that SCI disrupts gut community composition, interaction architecture, enteric innervation, and metabolic function—changes consistent with impaired barrier function and increased potential for gut-derived signals.

**Figure 5:**
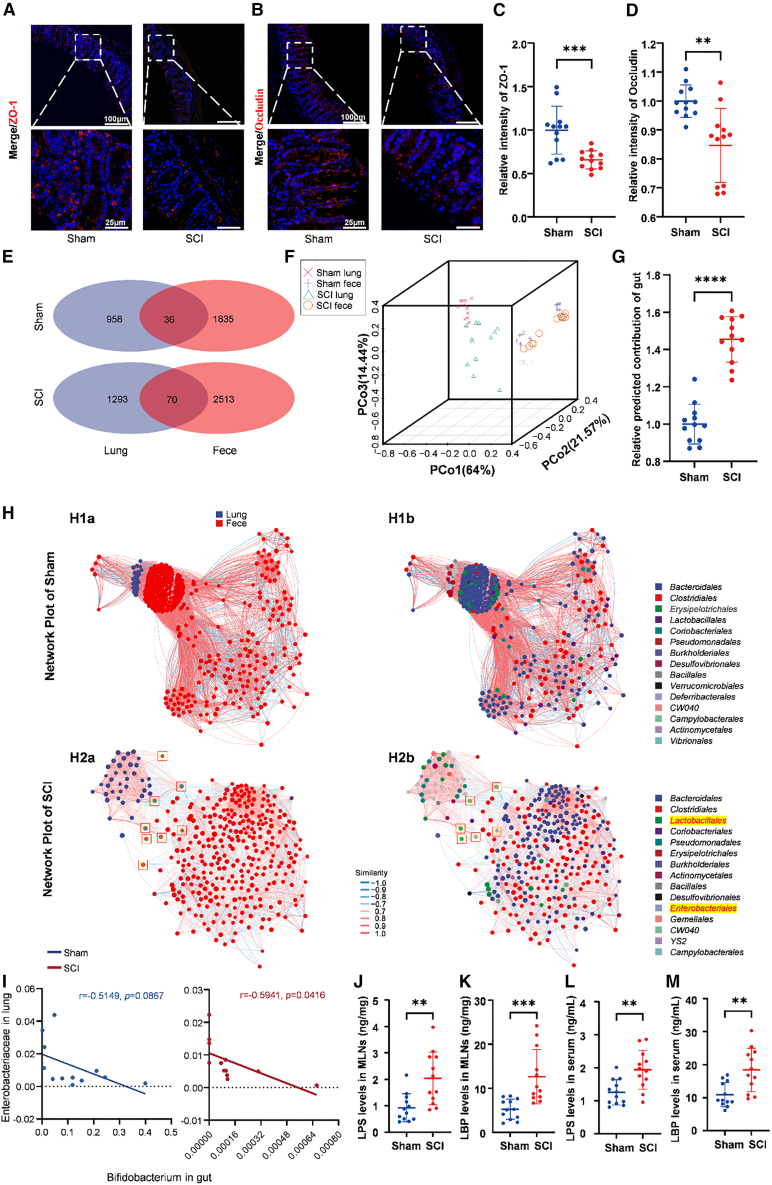
SCI weakens gut barrier, increases gut-lung microbiota overlap, and promotes systemic bacterial translocation (A) Representative images of immunofluorescence staining of ZO-1 (in red) in the colons of SCI and Sham mice (scale bars, 100 and 25 μm; *n* = 12). (B) Representative images of immunofluorescence staining of occludin (in red) in the colons of SCI and Sham mice (scale bars, 100 and 25 μm; *n* = 12). (C) Quantification of the relative fluorescence intensity of the intestinal ZO-1. ∗∗∗*p* < 0.001 for SCI versus Sham (2-tailed Student’s *t* test; mean ± SD; *n* = 12). (D) Quantification of the relative fluorescence intensity of intestinal occludin. (unpaired two-tailed *t* test; *p* < 0.01; *n* = 12 per group). (E) Venn diagrams illustrating shared ASVs between intestinal and lung microbiota in Sham and SCI mice (*n* = 12). (F) 3D PCoA of weighted UniFrac distances for all gut and lung samples showing closer gut-lung clustering in SCI (*n* = 12). (G) SourceTracker-estimated gut contribution to lung microbiota (unpaired two-tailed *t* test; ∗∗∗∗*p* < 0.0001; *n* = 12 per group). (H) Network plots illustrating relationships between intestinal and lung microbiota. (H1) Network plots of the Sham group colored by colonization site (H1a; blue = lung, red = feces) and by bacterial taxonomy (H1b). (H2) Network plots of the SCI group colored by colonization site (H2a) and by bacterial taxonomy (H2b), showing altered network topology (*n* = 12). (I) Pearson correlation analysis between gut *Bifidobacterium* and lung *Enterobacteriaceae* relative abundance (Pearson correlation; SCI: *p* = 0.0416, Sham: *p* = 0.0867; *n* = 12). (J) Quantification of LPS levels in MLNs (unpaired two-tailed *t* test; ∗∗*p* < 0.01; *n* = 12). (K) Quantification of LBP levels in MLNs (unpaired two-tailed *t* test; ∗∗∗*p* < 0.001; *n* = 10–12). (L) Quantification of LPS levels in serum (unpaired two-tailed *t* test; ∗∗*p* < 0.01; *n* = 12). (M) Quantification of LBP levels in serum (unpaired two-tailed *t* test; ∗∗*p* < 0.01; *n* = 12).

Given that *Bifidobacterium* supports anti-inflammatory tone and epithelial integrity,^35^ we next asked whether SCI promotes gut-to-lung microbial signals. Immunofluorescence of the tight junction proteins zonula occludens-1 (ZO-1) (*p* = 0.0006) and occludin (*p* = 0.001) showed reduced continuity and intensity in SCI intestines compared with Sham, consistent with barrier disruption after SCI (Figures 5A–5D).^36^^,^^37^

Cross-compartment overlap increased after injury. Venn diagram analysis of amplicon sequence variants (ASVs) showed more features shared by lung and gut within SCI mice than within Sham (SCI: 70; Sham: 36; Figure 5E). Ordination of all lung and gut samples using weighted UniFrac PCoA (3D visualization) revealed that, in SCI, paired lung and gut samples clustered more closely than in Sham, indicating increased cross-compartment similarity (Figure 5F). SourceTracker further estimated a higher gut contribution to SCI lungs, approximately 50% greater than in Sham (*p* < 0.0001; Figure 5G).^38^

To visualize interactions, we constructed a joint network of gut and lung taxa. In SCI, gut-associated orders such as *Lactobacillales* and *Enterobacterales* emerged as high-degree nodes in the lung subnetwork (Figure 5H). Across individuals, lung *Enterobacteriaceae* abundance was negatively correlated with gut *Bifidobacterium* in the SCI group (r = −0.5941, *p* = 0.0416), but not in the Sham group (r = −0.5149, *p* = 0.0867) (Figure 5I), consistent with loss of gut barrier function and altered gut-lung signaling.

To directly validate whether barrier dysfunction facilitates bacterial translocation, we quantified lipopolysaccharide (LPS) and LPS-binding protein (LBP) levels. Compared with Sham mice, SCI mice exhibited significantly elevated levels of LPS and LBP in both mesenteric lymph nodes (MLNs) (Figures 5J and 5K) and serum (*p* = 0.0024, *p* = 0.0008, *p* = 0.0026, *p* = 0.0025) (Figures 5L and 5M). Together, these data indicate that SCI weakens intestinal tight junctions, promotes systemic translocation of bacterial products, and increases gut-lung microbial overlap, supporting the hypothesis that gut-derived signals shape the injured lung microbiota.

### SCI microbiota transfer drives gut-lung convergence

**Figure 6:**
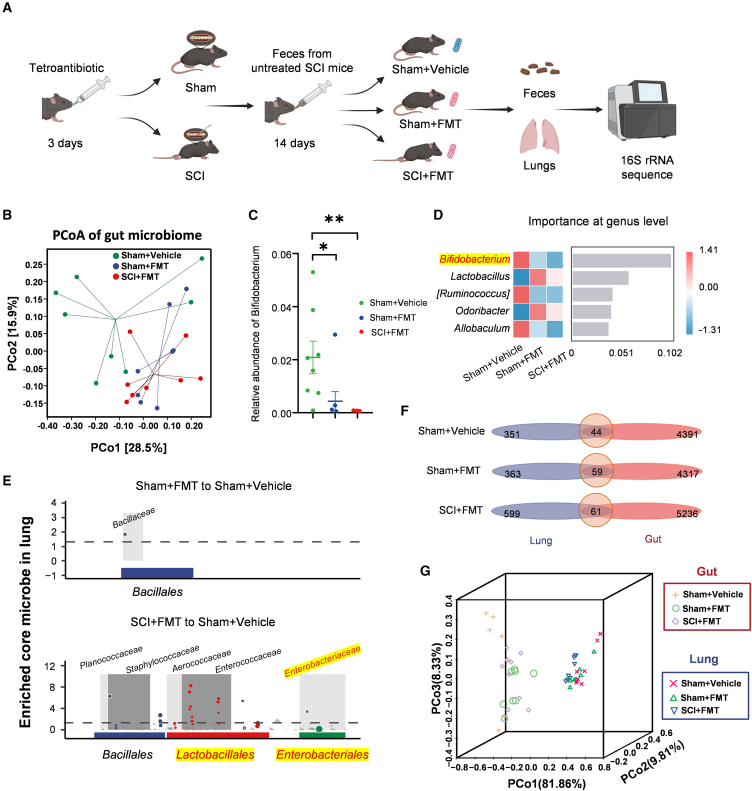
FMT from SCI donors reshapes gut communities and increases gut-lung convergence (A) Experimental flowchart. (B) PCoA of weighted UniFrac distances showing clustering of gut microbiota among Sham+Vehicle, Sham+FMT, and SCI+FMT (PERMANOVA, Sham+FMT vs. Sham+Vehicle, ∗*p* < 0.05; SCI+FMT vs. Sham+Vehicle, ∗*p* < 0.05; Sham+FMT vs. SCI+FMT, ns; *n* = 8 per group). (C) Relative abundance of *Bifidobacterium* (one-way ANOVA with Tukey’s HSD; mean ± SEM; ∗*p* < 0.05, ∗∗*p* < 0.01; *n* = 8 per group). (D) Random forest feature importance at the genus level distinguishing groups. (E) metagenomeSeq differential-abundance analysis identifying core taxa in Sham+FMT vs. Sham+Vehicle and in SCI+FMT vs. Sham+Vehicle. (F) Venn diagrams of shared ASVs between paired gut and lung samples within each group (Sham+Vehicle, Sham+FMT, SCI+FMT; *n* = 8 pairs per group). (G) 3D PCoA of weighted UniFrac distances for all gut and lung samples across groups (*n* = 8 per group).

To investigate whether the changes in gut and lung microbiota crosstalk post-SCI are driven by the intestinal microbiota, we performed FMT using fecal samples from SCI mice to colonize both Sham and SCI mice after antibiotic clearance of their original bacteria. We subsequently conducted 16S rRNA amplicon sequencing of the mouse lung microbiota and feces (Figure 6A).

Weighted UniFrac PCoA showed that FMT reshaped gut communities in Sham recipients. This was confirmed by PERMANOVA, which showed that the Sham+FMT group was significantly distinct from the Sham+Vehicle group (pseudo-F = 2.243, *p* = 0.041) but was not significantly different from the SCI+FMT group (pseudo-F = 0.551, *p* = 0.888), indicating that they clustered closely together (Figure 6B). At the genus level, *Bifidobacterium* was significantly reduced in both the Sham+FMT group (*p* = 0.0254) and the SCI+FMT group (*p* = 0.0055) when compared to the Sham+Vehicle group. Moreover, no significant difference was observed between the Sham+FMT and SCI+FMT groups (*p* = 0.7787) (Tukey’s multiple comparisons test; Figure 6C). Consistently, a genus-level random forest classifier ranked lower *Bifidobacterium* abundance among the top features distinguishing FMT-treated from vehicle-treated Sham recipients (Figure 6D).

We next asked how this gut reshaping influenced the lung microbiota. Differential abundance testing (metagenomeSeq) indicated that SCI+FMT lungs showed significant increases in families within the orders *Lactobacillales* (e.g., *Enterococcaceae*, adj. *p* = 8.77e-7) and *Enterobacterales* (e.g., *Enterobacteriaceae*, adj. *p* = 2.04e-7). In contrast, these taxa were not significantly enriched in Sham+FMT lungs, indicating that FMT in Sham recipients did not recapitulate this lung phenotype (Figure 6E). Nevertheless, cross-compartment overlap increased after FMT: Venn diagram analysis showed more shared ASVs between paired lung and gut samples in SCI+FMT (61 shared ASVs) than in Sham+Vehicle (44), with Sham+FMT also exceeding Sham+Vehicle (Figure 6F). A 3D PCoA of all lung and gut samples further visualized shorter gut-lung distances in Sham+FMT than in Sham+Vehicle (Figure 6G).

Together, these findings indicate that transferring SCI-associated microbiota reduces intestinal *Bifidobacterium* and increases gut-lung similarity, supporting a model in which gut-derived signals contribute to lung community changes after SCI.

### Increased *Bifidobacterium* reduces bacterial translocation in SCI mice

**Figure 7:**
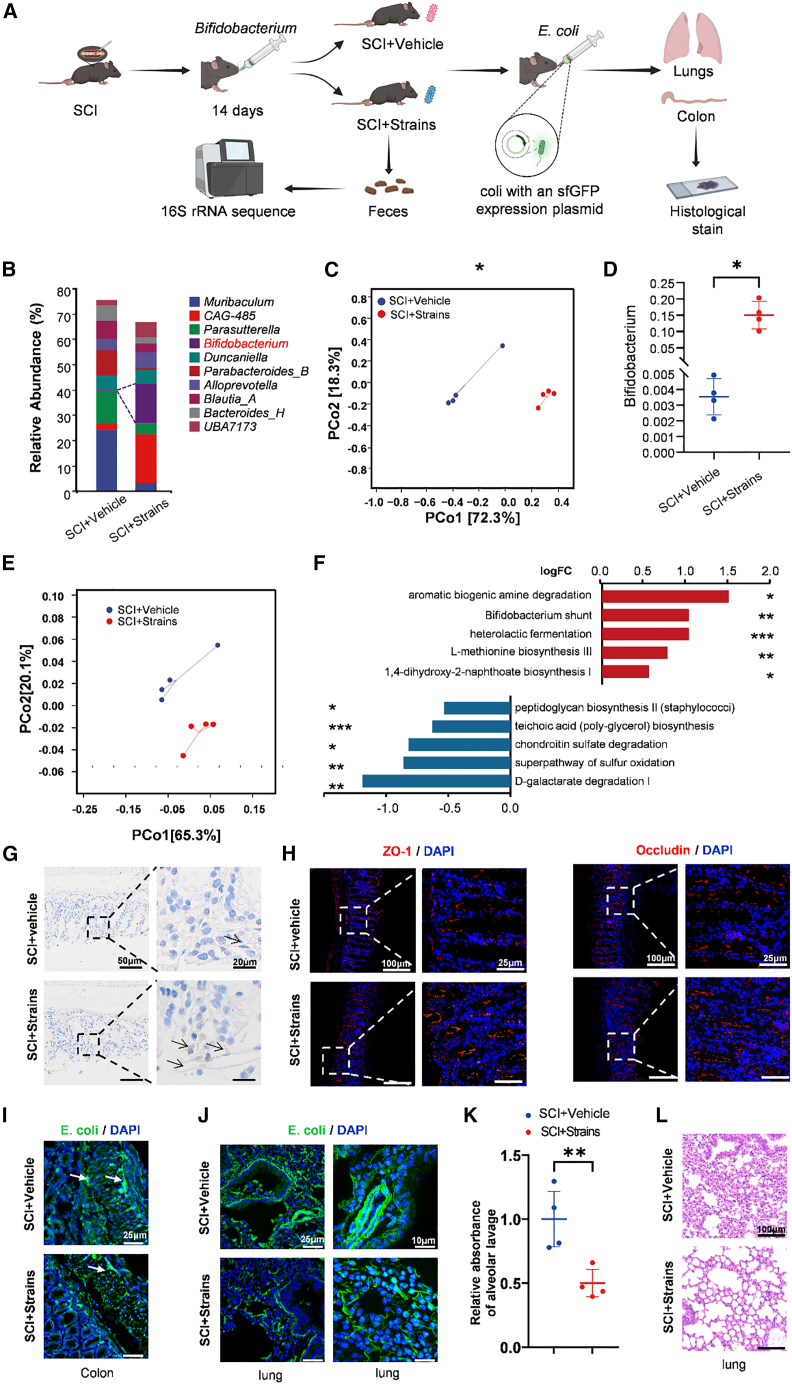
*Bifidobacterium* supplementation improves intestinal barrier and reduces gut-derived bacterial signals (A) Experimental flowchart. (B) Genus-level gut microbiota composition in SCI+Vehicle and SCI+Strains mice (*n* = 4 per group). (C) PCoA of weighted UniFrac distances showing clustering of gut microbiota in SCI+Vehicle vs. SCI+Strains (PERMANOVA, ∗*p* < 0.05; *n* = 4 per group). (D) Relative abundance of *Bifidobacterium* (genus level) (unpaired two-tailed *t* test; mean ± SD; ∗*p* < 0.05; *n* = 4 per group). (E) PCoA based on MetaCyc metabolic pathway abundances showing distinct functional clustering between the SCI+Vehicle and SCI+Strains groups. (F) Functional prediction of gut microbiota metabolic pathways (MetaCyc) using PICRUSt2. Bar plot shows the top 10 differentially abundant pathways. Red bars indicate pathways enriched in the SCI+Strains group, while blue bars indicate pathways enriched in the SCI+Vehicle group (two-sided Welch’s *t* test, ∗*p* < 0.05, ∗∗*p* < 0.01, ∗∗∗*p* < 0.001; *n* = 4 per group). (G) PGP9.5 immunohistochemistry of colon showing greater preservation of enteric neurons with supplementation (scale bars, 50 and 20 μm; *n* = 4 per group). (H) Immunofluorescence of ZO-1 and occludin (red; nuclei stained with 4′,6-diamidino-2-phenylindole [DAPI]) in colon (scale bars, 100 and 25 μm; *n* = 4 per group). (I) GFP-expressing *E. coli* (green) in colon (scale bars, 25 μm). (J) GFP-expressing *E. coli* in lung (scale bars, 25 and 10 μm; *n* = 4 per group). (K) BALF fluorescence (excitation 488 nm, emission 510–530 nm) showing reduced signal in SCI+Strains (mean ± SD; ∗∗*p* < 0.01; unpaired two-tailed *t* test; *n* = 4 per group). (L) Representative H&E-stained lung sections (scale bars, 100 μm).

After implicating *Bifidobacterium* in gut-lung interactions, we asked whether improving the intestinal milieu could mitigate dysbiosis after SCI. In the SCI+BL300 group, mice received oral gavage of *Bifidobacterium longum* BL300 (CGMCC 24068); SCI+Vehicle mice received PBS. We then gavaged GFP-expressing *Escherichia coli* (*E. coli*), family *Enterobacteriaceae*. Three days later, lungs and colon were collected for analysis (Figure 7A).

Taxonomic profiling showed a significant increase in the genus-level relative abundance of *Bifidobacterium* in SCI+BL300 versus SCI+Vehicle (Figure 7B). Weighted UniFrac PCoA indicated a compositional shift with supplementation (*p* = 0.027) (Figure 7C). Consistently, *Bifidobacterium* relative abundance was higher in SCI+BL300 (*p* = 0.0286) (Figure 7D).

To explore the functional implications of these compositional shifts, we performed predictive functional profiling using PICRUSt2 based on the MetaCyc database. PCoA of metabolic pathways revealed distinct functional clustering between the SCI+BL300 and SCI+Vehicle groups (Figure 7E). Differential abundance analysis identified a mechanistic reversal of the SCI-induced dysbiosis pattern (Figure 7F). Specifically, supplementation enriched pathways associated with short-chain fatty acids (SCFAs) production (e.g., *Bifidobacterium* shunt, heterolactic fermentation) and detoxification (aromatic biogenic amine degradation). Conversely, pathways linked to pathogen expansion (peptidoglycan biosynthesis II, teichoic acid biosynthesis) and mucus erosion (chondroitin sulfate degradation) were significantly downregulated.

To assess intestinal integrity, PGP9.5 immunohistochemistry demonstrated greater preservation of enteric neurons in SCI+BL300 (Figure 7G). Immunofluorescence showed partial restoration of the tight junction proteins ZO-1 and occludin (Figure 7H). In the intestine, submucosal *E. coli* signals were reduced in SCI+BL300 compared with SCI+Vehicle (Figure 7I). In the lungs, *E. coli* signals were likewise lower in SCI+BL300 (Figure 7J), and bronchoalveolar lavage fluid (BALF) fluorescence decreased (excitation 488 nm, emission 510–530 nm) (*p* = 0.009) (Figure 7K). H&E staining further demonstrated that the lungs of the SCI+Strains group did not exhibit pronounced inflammation compared with those of the SCI+Vehicle group (Figure 7L).

Together, these findings indicate that *Bifidobacterium longum* BL300 improves intestinal community composition and barrier features after SCI and reduces gut-derived bacterial signals to the lungs, consistent with limiting putative translocation or microaspiration and mitigating lung inflammation.

## Discussion

Prophylactic antibiotics have not produced meaningful reductions in pneumonia incidence or mortality in patients with SCI, reinforcing that respiratory complications remain a major driver of death and neurological decline after SCI.^3^ Known risk factors include respiratory muscle denervation, ventilatory weakness, and exposure to mechanical ventilation, while SCI-associated immunosuppression further predisposes patients to infection.^39^^,^^40^ Yet pneumonia also occurs in individuals in whom these factors are less pronounced, including those with lower thoracic injuries, implying additional mechanisms. We therefore examined the gut-lung axis and identified microbiome features associated with post-SCI respiratory vulnerability.

The long-standing notion of a sterile lung has been replaced by evidence for a low-biomass resident microbiota that shapes local immunity.^15^^,^^41^^,^^42^ Our data align with this paradigm. After SCI, we observed greater similarity between gut and lung microbial communities in both humans and mice, together with enrichment of taxa typical of gut/oral sources, including orders such as *Lactobacillales* and families such as *Enterobacteriaceae*, within lower-airway samples.^28^ A random forest classifier ranked *Lactobacillales* and *Enterobacteriaceae* among the top features discriminating SCI lungs from controls.

Source-tracking analyses supported cross-compartment convergence, and in mice, transection was followed by delayed pulmonary inflammation and compositional shifts. Several routes may contribute to this convergence. SCI-related autonomic dysfunction can impair esophageal and intestinal motility, altering the intestinal microbial niche. Concurrently, reduced expression of tight junction proteins (ZO-1 and occludin) indicates intestinal barrier compromise. Crucially, we detected significantly elevated levels of LPS and LBP in both MLNs and serum, providing biochemical evidence that barrier breach facilitates the systemic translocation of bacterial products to reshape the pulmonary microenvironment.

We also identified the genus *Bifidobacterium* as a potential modulator of gut-lung crosstalk. Intestinal *Bifidobacterium* was depleted after SCI and inversely correlated with lung *Enterobacteriaceae*. Biologically, *Bifidobacterium* helps maintain gut homeostasis by producing acetate and lactate, enhancing mucosal defenses.^43^ Our functional prediction analysis (PICRUSt2) mechanistically supported this, revealing that *Bifidobacterium* supplementation specifically enriched the “*Bifidobacterium* shunt” and “heterolactic fermentation” pathways—key drivers of SCFA production—while suppressing pathways associated with pathogen cell wall synthesis (e.g., peptidoglycan biosynthesis). This metabolic shift plausibly counteracts barrier dysfunction and the expansion of opportunistic Proteobacteria, consistent with the convergence pattern reproduced by FMT in our study.

Guided by this rationale, oral *Bifidobacterium longum* BL300 increased gut *Bifidobacterium* after SCI, partially corrected dysbiosis, and improved barrier markers (ZO-1 and occludin). Using GFP-labeled *E*. *coli* as a tractable *Enterobacteriaceae* surrogate, supplementation reduced submucosal intestinal and pulmonary GFP signals and attenuated lung inflammation. Prior studies similarly report probiotic-mediated modulation of inflammation in SCI models.^44^^,^^45^^,^^46^ Collectively, these data nominate microbiota-targeted therapy as a mechanistically distinct adjunct to current prevention and rehabilitation protocols, outline testable interventions and biomarkers, and underscore translational potential. *Bifidobacterium* strains are generally recognized as safe (GRAS) and are operationally feasible even in acute-phase SCI. Our work provides a rationale for clinical trials to test whether early, targeted probiotics or symbiotic can restore intestinal homeostasis and reduce the incidence or severity of pneumonia in patients with SCI.

Important limitations remain. Our data are consistent with bacterial translocation but do not prove the specific route (e.g., distinguishing hematogenous spread from microaspiration). Cross-sectional measurements lack kinetic resolution, and terminal histology cannot distinguish microaspiration from hematogenous spread or establish timing. Future studies should employ strain-resolved or barcoded tracking, longitudinal sampling, and integrated multi-omics to map routes and dynamics; apply rigorous contamination control to low-biomass respiratory samples; and optimize probiotic parameters (strain, dose, timing, and duration). Larger, multicenter cohorts are needed to assess safety and generalizability, compare probiotics with symbiotic/postbiotics or defined consortia, and determine whether modulation of the gut-lung axis can reduce respiratory complications in SCI.

### Limitations of the study

Important limitations remain in this study. First, while our data are consistent with bacterial translocation, they do not definitively prove the specific route of dissemination (e.g., distinguishing hematogenous spread from microaspiration). Second, our cross-sectional measurements lack kinetic resolution, and terminal histology cannot distinguish microaspiration from hematogenous spread or establish exact timing. Future studies should employ strain-resolved or barcoded tracking, longitudinal sampling, and integrated multi-omics to map routes and dynamics. Additionally, rigorous contamination control is essential for low-biomass respiratory samples, and probiotic parameters (strain, dose, timing, duration) require further optimization. Finally, larger, multicenter cohorts are needed to assess safety and generalizability, compare probiotics with symbiotic/postbiotics or defined consortia, and determine whether modulation of the gut-lung axis can effectively reduce respiratory complications in patients with SCI.

## Resource availability

### Lead contact

Further information and requests for resources and reagents should be directed to and will be fulfilled by the lead contact, Rong Xie (rongxie@fudan.edu.cn).

### Materials availability

This study did not generate new unique reagents.

### Data and code availability

• The 16S rRNA gene sequencing datasets generated during this study have been deposited at NCBI under BioProject IDs PRJNA1100250, PRJNA1100322, and PRJNA1100933. Accession numbers are listed in the key resources table.
• This paper does not report original code.
• Any additional information required to reanalyze the data reported in this paper is available from the lead contact upon request.

## Acknowledgments

We thank the National Center for Neurological Disorders; the Neurosurgical Institute of Fudan University; the Shanghai Clinical Medical Center of Neurosurgery; the Shanghai Key Laboratory of Brain Function and Restoration and Neural Regeneration, Huashan Hospital; and the Department of Endocrinology, The First Affiliated Hospital, Zhejiang University School of Medicine, for their support. We also thank Dr. Fengyun Zheng from the Core Facility of Shanghai Medical College, Fudan University, for support with slide image scanning and data analysis.

This study was supported by the National Natural Science Foundation of China (NSFC) (82071315, 82271342, and 82371396), Joint Funds for the Innovation of Science and Technology, Fujian Province (no. 2021Y9307 and no. 2023Y9114), Natural Science Foundation of Fujian Province (2023J01314), and the Tongren Science and Technology Bureau ([2025]67).

## Author contributions

Conceptualization, R.X. and Y.D.; data curation, Y.D. and X.C.; formal analysis, X.C., Z.Z., and X.X.; funding acquisition, R.X.; investigation, Y.D., X.C., H.D., Y.T., Z.Z., and X.X.; project administration, R.X., and Y.D.; supervision, R.X. and Y.D.; keywords: validation, Y.D., X.C., H.D., Y.T., Z.Z., and X.X.; visualization, Z.Z. and X.X.; writing – original draft, R.X. and Y.D.; writing – review & editing, R.X., Y.D., and X.C.

## Declaration of interests

The authors declare that no conflicts of interest exist.

## STAR★Methods

### Key resources table

**Table:** 

REAGENT or RESOURCE	SOURCE	IDENTIFIER
**Antibodies**		

Rabbit polyclonal anti-ZO-1	Abcam	Cat# ab216880; RRID: AB_2909434
Rabbit monoclonal anti-occludin	Abcam	Cat# ab216327; RRID: AB_2737295

**Bacterial and virus strains**		

GFP-expressing *Escherichia coli*	ATCC	Cat# 25922
*Bifidobacterium longum* BL300	CGMCC	Accession# 24068

**Chemicals, peptides, and recombinant proteins**		

Ampicillin	Ambrothia	Cat# AMP25B
Neomycin	Sigma-Aldrich	Cat# N6386
Gentamicin	Sigma-Aldrich	Cat# G1397
Vancomycin	Sigma-Aldrich	Cat# V2002
Amphotericin B	Duchefa	Cat# 1397-89-3

**Critical commercial assays**		

OMEGA Soil DNA Kit	Omega Bio-Tek	Cat# M5635-02
PicoGreen	Invitrogen	Cat# P7589
Mouse TNF-α ELISA Kit	Invitrogen	Cat# 88-7324-88
Mouse IL-1β ELISA Kit	Invitrogen	Cat# BMS6002-2TEN
Mouse IL-6 ELISA Kit	Invitrogen	Cat# 88-7064-88

**Deposited data**		

Raw and analyzed data	This paper	NCBI BioProject: PRJNA1100250, PRJNA1100322, PRJNA1100933

**Experimental models: Organisms/strains**		

Mouse: C57BL/6J, female	Fudan University	N/A

**Oligonucleotides**		

Primer: 16S V3-V4 Forward: ACTCCTACGGGAGGCAGCA	This paper	N/A
Primer: 16S V3-V4 Reverse: GGACTACHVGGGTWTCTAAT	This paper	N/A

**Software and algorithms**		

QIIME 2	(2019.4)	https://qiime2.org/
cutadapt	N/A	https://cutadapt.readthedocs.io/
DADA2	N/A	https://benjjneb.github.io/dada2/
MAFFT	N/A	https://mafft.cbrc.jp/alignment/software/
FastTree	N/A	http://www.microbesonline.org/fasttree/
metagenomeSeq	N/A	https://bioconductor.org/packages/release/bioc/html/metagenomeSeq.html
GraphPad Prism	GraphPad	v8.0.2/8.4.3
R	The R Foundation	https://www.r-project.org/
Image-Pro Plus	N/A	v7.0

### Experimental model and study participant details

#### Human participants

All procedures involving human participants were conducted in accordance with the Declaration of Helsinki. The study protocols were reviewed and approved by the Institutional Review Board (IRB) of Huashan Hospital, Fudan University (Approval No. KY2025-704). Written informed consent was obtained from all participants in the prospective cohort. For the retrospective cohort, the IRB approved a waiver of consent due to the de-identified nature of the data analysis.

#### Retrospective cohort

We conducted a retrospective analysis of electronic medical records from a cohort of 237 adult patients (≥18 years) who underwent thoracic spinal cord surgery at Huashan Hospital between 2019 and 2022. The cohort consisted of 202 patients with preserved thoracic cord function and 35 patients who developed postoperative thoracic cord dysfunction. In total, the cohort included 109 males and 128 females. The mean age was 46.0 ± 13.0 years for the SCI group and 48.0 ± 13.4 years for the non-SCI group. We analyzed the influence of sex on study outcomes and found that baseline characteristics of sex (P=0.170) and age (P=0.396) were comparable between the two groups, indicating no significant sex-based bias in group allocation. All participants were of Han Chinese ancestry. Information on socioeconomic status was not collected. Key data extracted included demographics, presenting symptoms, American Spinal Injury Association (AIS) impairment scale grades, and relevant physiological parameters. Exclusion criteria were the presence of chronic inflammatory or infectious diseases, active malignancy, known immunosuppressive conditions, use of immunosuppressive medications, pre-existing neutropenia or lymphopenia, and recent use of systemic antibiotics or adrenergic antagonists.

#### Prospective cohort

We prospectively enrolled a cohort of 14 patients undergoing thoracic spinal cord surgery who had no pre-existing respiratory disease. This cohort included 7 males and 7 females, with a mean age of 36.4 ± 15.6 years (range: 15–61). All participants were of Han Chinese ancestry. Postoperatively, participants were categorized as either controls (n=7) or iatrogenic SCI (n=7). One week after surgery, sputum samples were collected for 16S rRNA gene sequencing.

#### Animal model

Female C57BL/6J mice (7–10 weeks old) were obtained from Fudan University and housed under specific pathogen-free (SPF) conditions. Mice were group-housed (up to 6 per cage) in a facility with a 12-hour light/dark cycle at 22 ± 2 °C and had *ad libitum* access to food and water. Female mice were exclusively selected for this study to minimize mortality resulting from urinary tract infections commonly associated with SCI models; therefore, the influence of sex on SCI outcomes was not evaluated in this animal cohort. All animal procedures were conducted in accordance with the ARRIVE guidelines and were approved by the Animal Welfare and Ethics Review Committee of the Department of Laboratory Animal Science, Fudan University (Approval No. 2022JS-Huashan-316).

### Method details

#### Spinal cord transection and perioperative care

Female C57BL/6J mice were selected to reduce post-SCI mortality from urinary tract infection. Under inhalational anesthesia, mice were placed on a heated pad (target 36 °C). After dorsal shaving and antisepsis, a midline incision was made, paraspinal muscles at T8–T10 were blunt-dissected, and a T9 laminectomy was performed, followed by complete spinal cord transection. No systemic antibiotics were administered to avoid confounding spontaneous lung findings. Animals received perioperative analgesia and bladder care per institutional SOPs. Randomization and blinded outcome assessment were implemented for histology and image quantification.

#### Sample collection

At the designated time points (3, 7, 14, and 28 days post-injury), mice were anesthetized and sacrificed. For microbiota analysis, fresh fecal pellets were collected from the colon under sterile conditions and immediately snap-frozen in liquid nitrogen. Lung tissues were harvested aseptically; one portion was snap-frozen for DNA extraction and cytokine analysis, while the other was fixed in 4% neutral-buffered formalin for histological examination. Peripheral blood was collected via the retro-orbital sinus, and serum was isolated by centrifugation for subsequent analysis.

#### DNA extraction

DNA was extracted from samples using the OMEGA Soil DNA Kit following the manufacturer’s instructions. The extracted DNA was stored at −20 °C. DNA concentration and purity were measured using a Nanodrop NC2000 spectrophotometer and confirmed by agarose gel electrophoresis.

#### 16S rRNA gene amplicon sequencing

The V3–V4 hypervariable region of the 16S rRNA gene was amplified using primers 338F (5'-ACTCCTACGGGAGGCAGCA-3') and 806R (5'-GGACTACHVGGGTWTCTAAT-3') with 7-bp barcodes. PCR was performed using FastPfu DNA polymerase with the following cycling conditions: 98 °C for 5 min; 25 cycles of 98 °C for 30 s, 53 °C for 30 s, and 72 °C for 45 s; and a final extension at 72 °C for 5 min. Amplicons were purified, quantified with PicoGreen, pooled equimolarly, and sequenced on an Illumina NovaSeq 6000 platform.

#### Sequence and microbiome analysis

Raw sequencing reads were processed using QIIME 2 (2019.4). After demultiplexing and primer trimming with cutadapt, sequences were processed with DADA2 to denoise, merge, and remove chimeras, generating amplicon sequence variants (ASVs). ASVs were aligned using MAFFT, and a phylogenetic tree was constructed with FastTree. Taxonomy was assigned against the SILVA database (release 132). Alpha diversity (Chao1, Shannon) and beta diversity (weighted UniFrac, Bray–Curtis) were calculated and visualized with PCoA. PERMANOVA was used to test for group separation. LEfSe and random forest analyses were used to identify discriminative taxa.

#### Blood and cytokine analyses

Peripheral blood (50 μL) was collected via the retro-orbital sinus into tubes containing 100 μL 10 mM EDTA at 3, 7, 14, and 28 dpi or from Sham controls, and analyzed on a hematology analyzer (Mairi BC-2800 Vet). Lung tissue lysates were stored at −80 °C. Cytokines were measured using mouse ELISA kits for TNF-α, IL-1β, and IL-6 according to manufacturer protocols.

#### Histology and immunofluorescence

Tissues were fixed in neutral-buffered formalin for ∼12 h, paraffin-embedded, sectioned at 4 μm, and stained with hematoxylin and eosin (H&E) (Wuhan Servicebio). Slides were anonymized and read by a board-certified pathologist blinded to group allocation. Paraffin-embedded intestinal sections (4 μm) were equilibrated in 0.1 M Tris-buffered saline, blocked with 10% normal goat serum in PBS (1 h), and incubated with primary antibodies to ZO-1 (rabbit polyclonal, 1:100, Abcam ab216880) and occludin (rabbit monoclonal, 1:100, Abcam ab216327) for 1 h, followed by fluorophore-conjugated secondary antibodies and DAPI. Slides were coverslipped with glycerol-based medium and imaged by fluorescence microscopy (Olympus SLIDEVIEW VS200 research slide scanner). Relative fluorescence intensity was quantified in Image-Pro Plus 7.0 under identical exposure settings; investigators were blinded to group.

#### Antibiotic-mediated depletion of intestinal bacteria

For microbiota depletion, mice received drinking water containing ampicillin (1 g/L; Ambrothia AMP25B), neomycin (1 g/L; Sigma N6386), gentamicin (1 g/L; Sigma G1397), metronidazole (1 g/L; Sigma M3761), vancomycin (0.5 g/L; Sigma V2002), and amphotericin B (0.1 g/L; Duchefa 1397-89-3) for 3 days starting 5 days before inoculation. Bottles were changed daily; intake was monitored.

#### Fecal sample collection and fecal microbiota transplantation (FMT)

Fresh feces from SCI donors were homogenized (≈20 mg in 2 mL PBS), briefly centrifuged (1,500 rpm, 3 min), and the supernatant (∼1 mL) collected. Recipients received 200 μL by oral gavage three times per week for 2 weeks starting after surgery (through 14 dpi). Fecal slurries were prepared fresh on ice and used within 1 h; aliquots were frozen at −80 °C as needed for batch consistency.

#### Probiotic supplementation and GFP–*E. coli* challenge

*Bifidobacterium longum* BL300 (CGMCC 24068) was cultured anaerobically at 37 °C on MRS medium supplemented with 0.5% L-cysteine. SCI mice received oral gavage of 2×10^8^ CFU in 200 μL PBS three times weekly for 2 weeks. Mice were then gavaged with GFP-expressing *E. coli* (ATCC 25922; 1×10^8^ CFU in 200 μL PBS). Three days later, mice were euthanized for tissue analyses.

### Quantification and statistical analysis

Analyses were performed in GraphPad Prism v8.0.2/8.4.3 and R. Data are mean ± SEM unless stated. Statistical significance was set at two-tailed P<0.05 with multiple-testing correction where applicable (FDR). Pre-specified outlier handling used the ROUT method (Q=1%) and is reported with the number removed. Between-group comparisons used the Mann–Whitney U test or unpaired t test (after normality checks) and one-way ANOVA with Tukey’s post hoc test for >2 groups. Beta-diversity differences were assessed by PERMANOVA. Effect sizes (e.g., R^2^, Cohen’s d, or rank-biserial r) and exact n values are reported in figure legends. All analyses were performed with investigators blinded to group assignments. Detailed statistical information, including exact p values and sample sizes, is provided in Table S3.
