# Supplementary material for: Gut-lung axis perturbation and Bifidobacterium potential after spinal cord injury in humans and mice
Source: iScience. 2026 Jan 9;29(2):114655. doi: 10.1016/j.isci.2026.114655 (PMC12876623; doi:10.1016/j.isci.2026.114655)
Supplement: Document S1. Figures S1 and S2 and Tables S1–S3 [file mmc1.pdf]

iScience, Volume *29*

Supplemental information

Gut-lung axis perturbation and *Bifidobacterium* potential after spinal cord injury in humans and mice

Yuanqing Ding, Xingyu Chen, Yiming Tao, Haoru Dong, Zezhen Zhang, Xiao Xiao, Gong Chen, Xiaomu Li, and Rong Xie

Supplementary Materials:

Supplementary Table 1: Baseline characteristics and clinical data

Baseline characteristics and clinical data		With SCI	W/o SCI	P-value
Age: Mean ± SD		46.0 ± 13.0	48.0 ± 13.4	0.396
Gender. Male/total n (%)		13/ 35(37.1)	96/202(47.5)	0.170
Pneumonia associated clinical symptoms n(%)	Neurosurgery Level ： T1-T4	4/35(11.4)	12/202(6.0)	0.009
	Neurosurgery Level ： T5-T8	4/35(11.4)	10/202(5.0)	
	Neurosurgery Level ： T9-T12	5/35(14.3)	13/202(6.4)	

Distribution of baseline characteristics and clinical characteristics as occurring during follow up. The American Cardiothoracic Society guidelines define standard clinical diagnostic criteria for hospital-acquired pneumonia, which include at least two of the following three findings: fever, leukocytosis or leukopenia, and septic tracheal secretions.For statistical comparison between the groups (with SCI-associated pneumonia (SCIAP) or w/o SCI-AP) the Student's t-test test was used for age, the Chi-square test was applied for all other variables. Differences in patient numbers within the groups result from missing data for the respective variables. Abbreviations: SD = standard deviation，SCI=spinal cord injury.

Supplementary Table 2: Distribution of pneumonia incidence in different surgical levels

Level of neurosurgery	T1-T4 n / total n (%)	T5-T8 n / total n (%)	T9-T12 n / total n (%)	P-value
SCI-APS	4/10(40)	4/9(44.4)	5/16(31.3)	0.822

Distribution of pneumonia incidence has no significant difference in different surgical levels. For statistical comparison between the groups (with SCI-associated pneumonia symptoms (SCI-APS) or w/o SCI-APS) the Chi-square test was applied for all other variables. . Abbreviations: SD = standard deviation.

Supplementary Table 3: The statistical information

Figure Panel	Groups Compared	Statistical Test	Exact P-value	N (Biological Replicates)
**Figure 1**				
Fig 1B	Patients w/ vs w/o SCI (Pneumonia symptoms)	Chi-square test	p = 0.009	w/o SCI n=202; SCI n=35
Fig 1C	Pneumonia by Level (T1-4 vs T5-8 vs T9-12)	Chi-square test	p = 0.822	n=10, 9, 16
Fig 1D	Human Sputum Microbiota (Control vs SCI)	PERMANOVA (Weighted UniFrac)	p = 0.008	n=7 per group
**Figure 2**				
Fig 2C	Histology Score (Sham vs SCI) at 3, 7, 14, 28 dpi	Mann-Whitney test (at each timepoint)	p = 0.0022 (all timepoints)	n=6 per group
Fig 2E	CD68+ Cell Count (Sham vs SCI) at 14, 28 dpi	Unpaired t-test	14dpi: p = 0.0011; 28dpi: p = 0.0118	n=6 per group
Fig 2F	Lung Cytokines (SCI: 3dpi vs 14dpi)	Unpaired t-test	TNF-a: p=0.0481; IL-1b: p=0.0461; IL-6: p=0.0443	n=6 per group
**Figure 3**				
Fig 3D	Alpha Diversity (Chao1, Observed, Coverage)	Kruskal-Wallis test	Chao1: p=0.0015; Observed: p=0.021; Coverage: p=0.00031	n=12 per group
Fig 3E	Lung Microbiota Beta Diversity (Sham vs SCI)	PERMANOVA (Weighted UniFrac)	p = 0.001	n=12 per group
**Figure 5**				
Fig 5C	ZO-1 Intensity (Sham vs SCI)	Student's t-test (two-tailed)	p = 0.0006	n=12 per group
Fig 5D	Occludin Intensity (Sham vs SCI)	Unpaired t-test	p = 0.001	n=12 per group
Fig 5G	SourceTracker Gut Contribution	Unpaired t-test	p < 0.0001	n=12 per group
Fig 5I	Correlation (Lung Entero vs Gut Bifido)	Pearson correlation	SCI: p=0.0416; Sham: p=0.0867	n=12 per group
Fig 5J	LPS in MLNs (Sham vs SCI)	Unpaired t-test	p = 0.0024	n=12 per group
**Figure 6**				
Fig 6B	Gut Beta Diversity (Sham+FMT vs Sham+Veh)	PERMANOVA	p = 0.041	n=8 per group
Fig 6B	Gut Beta Diversity (Sham+FMT vs SCI+FMT)	PERMANOVA	p = 0.888	n=8 per group

Fig 6C	Bifidobacterium Abundance	One-way ANOVA (Tukey's post hoc)	Sham+FMT vs Sham+Veh: p=0.0254; SCI+FMT vs Sham+Veh: p=0.0055; Sham+FMT vs SCI+FMT: p=0.7787	n=8 per group
**Figure 7**				
Fig 7C	Gut Beta Diversity (SCI+Veh vs SCI+Strains)	PERMANOVA	p = 0.027	n=4 per group
Fig 7D	Bifidobacterium Abundance	Unpaired t-test	p = 0.0286	n=4 per group
Fig 7F	Metabolic Pathways (PICRUSt2)	Welch's t-test (two-sided)	$p < 0.05$	n=4 per group
Fig 7K	BALF Fluorescence	Unpaired t-test	p = 0.009	n=4 per group

Supplemental Figure 1

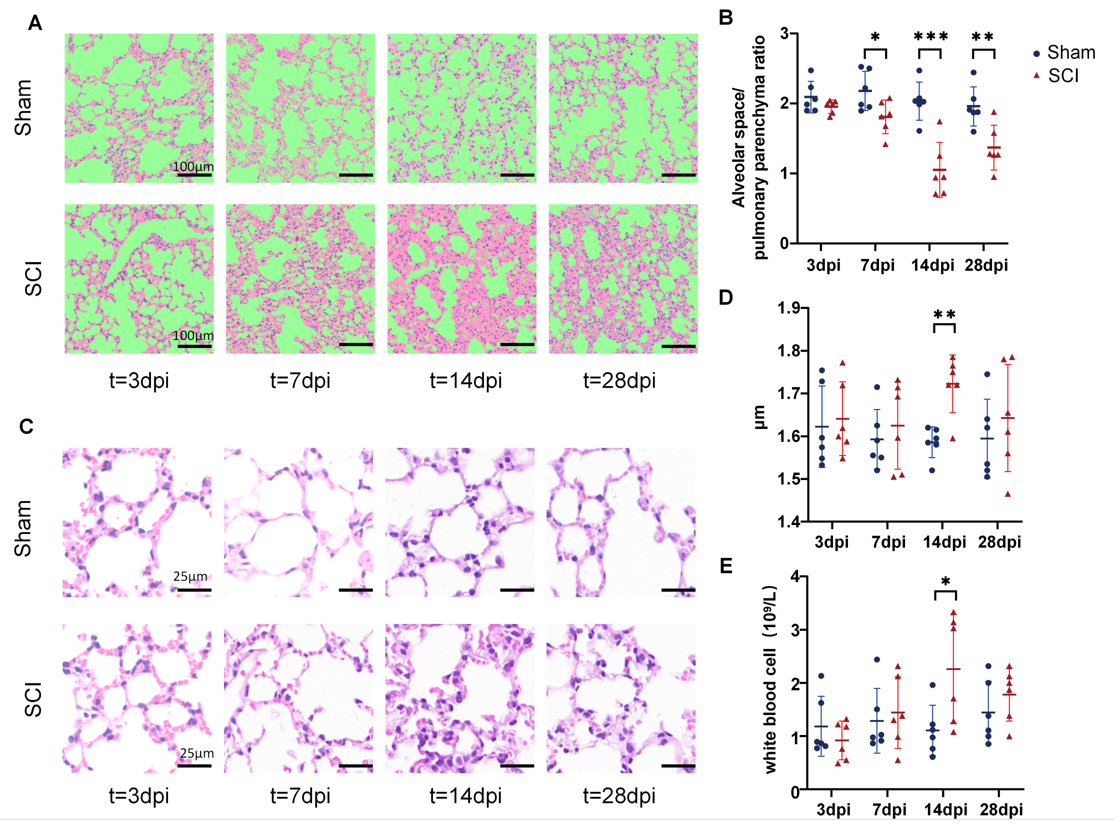

Supplemental Figure 1: (A) The selection of tissue (red signal) and alveolar (white signal) areas from the image of an H&E slide. The tissue areas (red signal) were defined with hue (0-255), saturation (0-255), and intensity (165-255), while the alveolar areas (white signal) were set with hue (0-255), saturation (0-255), and intensity (0-164). The selected areas were highlighted in green. (B) Quantification of the degree of alveolar space/pulmonary parenchyma ratio. $*P < 0.05$, $**P < 0.01$; $***P < 0.001$ for Sham versus SCI (2-tailed Student's t test; n=6, per time point; Error bars represent mean ± SD). (C) Representative H&E-stained cross sections of the alveoli from the SCI and Sham groups. (D) Quantification of the thickness of alveolar epithelial cells. $**P < 0.01$ for Sham versus SCI (2-tailed Student's t test; n=6, per time point; Error bars represent mean ± SD). (E) White blood cell count in bronchoalveolar lavage fluid. $*P < 0.05$ for Sham versus SCI (2-tailed Student's t test; n=6, per time point; Error bars represent mean ± SD).

Supplemental Figure 2

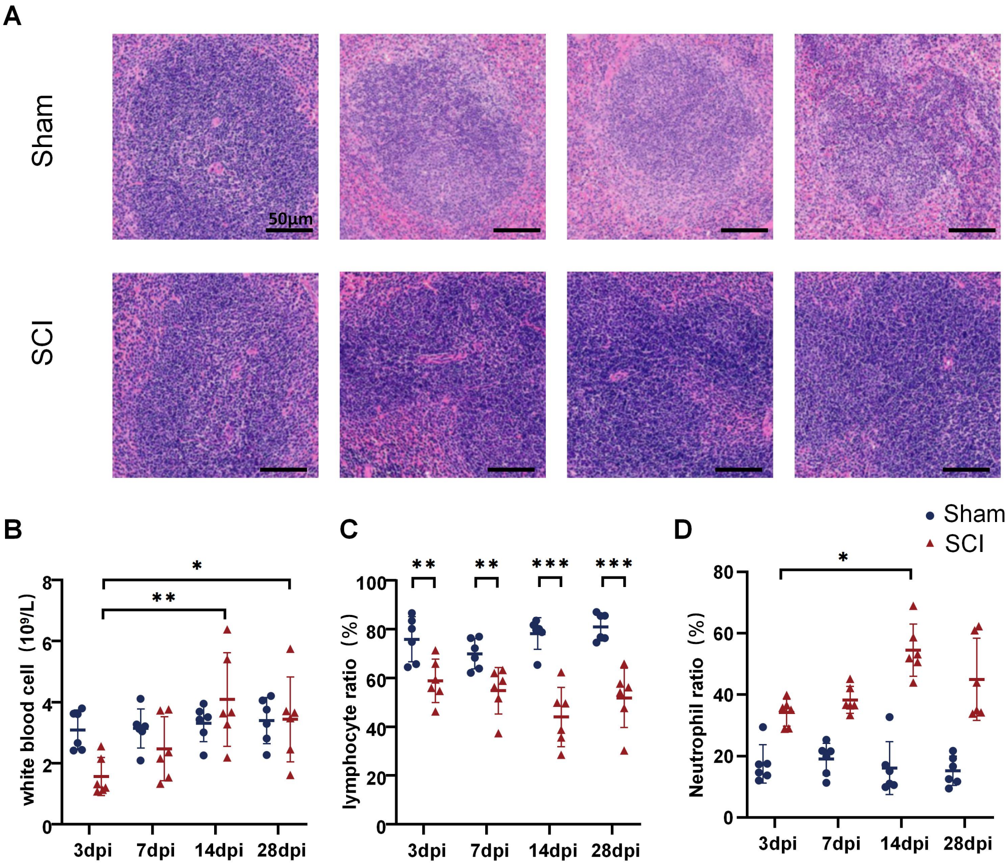

Supplemental Figure 2: (A) Representative H&E-stained cross sections of the splenic lymphoid hyperplasia from the SCI and Sham groups. (B-D) Complete blood count (CBC) of mice from the SCI and Sham groups. (B) Quantification of the white blood cell count. * $P < 0.05$ for SCI (28 dpi) versus SCI(3 dpi); ** $P < 0.01$ for SCI (14 dpi) versus SCI(3 dpi), (2-tailed Student's t test; n=6, per time point; Error bars represent mean ± SD). (C) Quantification of the lymphocyte percentage in the CBC of mice. ** $P < 0.01$, *** $P < 0.001$ for SCI versus Sham 2-tailed Student's t test; n=6, per time point; Error bars represent mean ± SD). (D) Quantification of the neutrophil percentage in the CBC of mice. * $P < 0.05$ for SCI (14 dpi) versus SCI(3 dpi), (2-tailed Student's t test; n=6, per time point; Error bars represent mean ± SD).
